# Advances in Lipidomics for Cancer Biomarkers Discovery

**DOI:** 10.3390/ijms17121992

**Published:** 2016-11-28

**Authors:** Francesca Perrotti, Consuelo Rosa, Ilaria Cicalini, Paolo Sacchetta, Piero Del Boccio, Domenico Genovesi, Damiana Pieragostino

**Affiliations:** 1 Department of Neurosciences and Imaging, University “G. d’Annunzio” of Chieti-Pescara, 66100 Chieti, Italy; francescaperrotti@libero.it (F.P.); c.rosa155@gmail.com (C.R.); d.genovesi@unich.it (D.G.); 2 Radiation Oncology Unit, SS Annunziata Hospital, 66100 Chieti, Italy; 3 Department of Pharmacy, University “G. d’Annunzio” of Chieti-Pescara, 66100 Chieti, Italy; ilariacicalini1@gmail.com (I.C.); p.delboccio@unich.it (P.D.B.); 4 Department of Medical Oral and Biotechnological Sciences, University “G. d’Annunzio” of Chieti-Pescara, 66100 Chieti, Italy; ps@unich.it; 5 Analitical Biochemistry and Proteomics Unit, Research Centre on Aging (Ce.S.I), University “G. d’Annunzio” of Chieti-Pescara, 66100 Chieti, Italy

**Keywords:** cancer, lipidomics, biomarkers, prognostic factors, diagnostic factors

## Abstract

Lipids play critical functions in cellular survival, proliferation, interaction and death, since they are involved in chemical-energy storage, cellular signaling, cell membranes, and cell–cell interactions. These cellular processes are strongly related to carcinogenesis pathways, particularly to transformation, progression, and metastasis, suggesting the bioactive lipids are mediators of a number of oncogenic processes. The current review gives a synopsis of a lipidomic approach in tumor characterization; we provide an overview on potential lipid biomarkers in the oncology field and on the principal lipidomic methodologies applied. The novel lipidomic biomarkers are reviewed in an effort to underline their role in diagnosis, in prognostic characterization and in prediction of therapeutic outcomes. A lipidomic investigation through mass spectrometry highlights new insights on molecular mechanisms underlying cancer disease. This new understanding will promote clinical applications in drug discovery and personalized therapy.

## 1. Introduction

Cancer is one of the most relevant public health problems, as it monopolizes a large part of the healthcare budget in many countries [1]. Despite significant improvements in cancer diagnoses and treatments obtained over the past few decades, the early detection of cancer through diagnostic, prognostic and predictive biomarkers represents one of the most promising research fields for identifying early-stage cancer and personalizing therapies. Biomarkers could be improved both for diagnosing the disease as well as predicting drug efficacy and classifying tumoral stages. However, the lack of reliable biomarkers complicates the personalized care of cancer patients. The heterogeneity of the various tumors is related to the variability of the oncogenic event [2]. Moreover, the same histology has different characteristics in terms of genetic background, metabolism, motility, variable proliferation and metastatic potential that could determine therapeutic failure. This heterogeneous nature of cancer can explain the lack of a specific biomarker with 100% diagnostic accuracy. Therefore, the attempt of metabolomic, proteomic and lipidomic approaches is to provide a better molecular definition of neoplasms [3].

Lipids play many essential roles in cellular functions, such as survival, proliferation and death, since they are involved in chemical-energy storage, cellular signaling, cell membranes, and cell–cell interactions in tissues. These cellular processes are strongly related to carcinogenesis pathways, particularly to transformation, progression, and metastasis. Specifically, several cellular functions, such as signal transduction, post-translational modifications, homeostasis, adhesion, migration, apoptosis, neurotransmission, vesicular trafficking and energy storage are regulated by phospholipids (PLs) [4,5,6,7]. Therefore, bioactive lipids play an important role in many biological process, in particular their composition is altered in many neoplastic diseases [8]. Several previous research groups have investigated lipid alterations in cancer cells to elucidate the disease and discover potential biomarkers [9]. For example, eicosanoids play a role in neoplastic transformation of cancer cells, generated effects in proliferation, motility, migration, invasion, apoptosis, metastasis and angiogenesis. Prostaglandin E2 PGE2 effects can be regulated by T cells, and this can provide tumor cell evasion [10]. After genomics and proteomics, lipidomics was first employed in 2003 as a metabolomic approach to investigate the qualitative and quantitative profile of the lipid components from serum, plasma, tissue, cells and organisms [11]. Lipidomic analyses aim to provide a comprehensive identification and quantification of all lipids and to characterize their interactions with other lipids, proteins or gene expression [12].

Thus, lipidomics is an emerging approach in tumor characterization for detecting and classifying neoplastic cells or tissues, as well as differentiating between a neoplasmic and normal environment. Moreover, this approach allows the evaluation of anticancer treatment, in terms of responsivity or resistance, and can be applied to the discovery of new tumor biomarkers [9]. Integration of lipidomic strategies in cancer research could generate new opportunities to obtain insights into diagnosis, prognosis and prediction of tailored therapies [13,14].

## 2. Lipid Classes

There are different classes of lipids related to cancer, divided into eight categories: fatty acids or acyls (FAs), glycerophospholipids (GPLs), glycerolipids (GLs), prenol lipids (PRLs), saccharolipids (SCLs), sphingolipids (SLs) and sterol lipids (STLs) [15]. Structurally, FAs are simple lipids, whereas GPLs, GLs, SLs, and STLs are complex [16,17,18,19,20,21]. The enzymatic or non-enzymatic oxidation of arachidonic acid or other polyunsaturated fatty acids (PUFAs) generates eicosanoids, which are signaling molecules widely studied in cancer [22]. The GPLs, including lysophospholipids (LPLs), and SLs are defined as polar lipids. GPLs compose bio-cellular membranes; LPLs act as ligands for numerous signaling pathways [23]. The polar lipids (GPLs, LPLs and SLs) are so involved in critical cell functions and they are the principal lipid class studied in lipidomic research. Based on different types of polar head groups, chain length of FAs, number of acyl-chains, degree of saturation in the acyl-chain, and number of double bonds, GPLs can be differentiated into several subclasses [24]: phosphatidic acid (PA), phosphatidylcholines (PC), phosphatidylethanolamine (PE), phosphatidylglycerol (PG), phosphatidylinositol (PI) and the last is phosphatidyloserine (PS). Bio-membranes are particularly rich in PC and PE. Similarly, SLs are essential bioactive compounds of cellular membrane, with important biological functions. SLs include ceramides, cerebrosides, gangliosides, sphingomyelins (SMs) and sulfatides (STs). SL structure is formed by a sphingoid base backbone; the head group attached to the primary hydroxyl group, *N*-acyl group and sphingoid-base backbone determines a change in structure of SLs [25]. The main classes of PLs are summarized in Table 1.

## 3. Sample Preparation

An advanced lipidomic approach combines the latest mass spectrometry (MS) technology and bioinformatics tools with traditional procedures for sample preparation and lipid extraction [26].

The extraction procedures for both neutral and polar lipids were developed at the end of the 1950s [27,28] and are still widely used. PL extraction methods, for example, were improved, starting from the “Bligh and Dyer” method and up to methods with higher recovery and efficiency, such as mixture of methanol/chloroform/methyl tert-butyl ether (MTBE) or ultrasound-combined extraction [29]. An update of used extraction procedures are described in the review of Seppanen-Laakso et al. [26].

## 4. Analytical Technology in Lipidomics

To date, lipidomic analytical strategies are applied to a wide variety of biological samples such as blood, plasma, serum, cerebrospinal fluid, urine and biological tissue derived from animal models or clinical patients [30]. The choice of the most appropriate analytical technique is based on the characteristics of the biological sample and the chemical properties of the targeted lipids. Nuclear magnetic resonance (NMR) and MS have become powerful tools for phospholipid structure identification [31]. Due to the structural diversity of the different phospholipid classes, the development of analytical methods for lipidomics is constantly changing. Lipid research has made great progress through the coupling of MS with chromatographic separations, especially by the use of soft ionization techniques as matrix-assisted laser desorption and ionization (MALDI) and electrospray ionization (ESI) [32,33].

An emerging approach in lipidomic application is infrared or Raman spectroscopy, offering a molecular signature, suitable for in vivo or in vitro diagnostics. Raman spectroscopy is a type of vibrational spectroscopy that allows to gain structural information through the scattering of incident light [34]. The techniques offer a wide range of applications from basic sciences to endoscopic probes equipped with micro-spectrometers for analysis of biofluids [35]. These probes contain both a source of light and a detector relating a signal back to the computer. Applications were realized to colonic and esophageal tissue directly in patients [36]. Blood [37] and urine [38] were also easily analyzed since the aqueous solution shows minimal background.

### 4.1. Electrospray Ionization Mass Spectrometry (ESI-MS/MS)

The introduction of electrospray ionization mass spectrometry (ESI-MS/MS) to analyze intact phospholipids provides specific information about the polar head group through the product-ions. The data, obtained as mass/charge ratio (*m*/*z*), provide a great deal of information already by MS analysis, if sufficiently accurate. The possibility of fragmenting by MS/MS mode, in which lipid molecules were fragmented thanks to collision gas, allows for the attainment of important structural information. The analytes may be revealed through different types of analyzer as time-of-flight (TOF) or triple quadrupole [39].

Direct-injection MS is an analytical method without previous chromatographic separation of lipids; it is a less time consuming and more reproducible than other methods, but it has the disadvantage of ion suppression.

Instead, the coupling of chromatography with MS carries a significant amount of information for the complex samples lipidome. Gas-chromatography (GC) is used for the separation of neutral lipids, such as triglycerides or cholesterol and cholesteryl esters, while high performance liquid chromatography (HPLC) is a very useful technique for the separation of polar lipids as phospholipids in their subclasses (PC, SM, PE, PG, PS, PI). The HPLC–ESI/MS strategy can enhance sensitivity and accuracy for low abundance lipids and was generally applied to targeted and un-targeted lipidomics. Recently, thanks to the development of ultra-high performance liquid chromatography (UHPLC), the resolution is greatly increased, in turn reducing ion suppression [9].

### 4.2. Matrix-Assisted Laser Desorption/Ionization Time-of-Flight/Mass Spectrometry (MALDI-TOF/MS)

MALDI mass spectrometry appeared as a fast and sensitive approach for lipidomics, since it tolerates sample impurities to a relatively high extent and offers clean mass spectra without major fragmentation of the analytes, as Fuchs et al. have well reviewed [40]. This advantage of being able to analyze a sample directly on the tissue, as with the MALDI-IMS (imaging MS) approach, allows visualization of the spatial distribution of phospholipid in tumor tissue.

Furthermore, the MALDI-MS technology is widely applied to biological fluid investigation for biomarker discovery. In this approach, the chromatographic separation of a lipid mixture can be achieved off-line by solid phase extraction (SPE), thin layer chromatography (TLC) and HPLC [41].

Various matrices have been used for MALDI-MS analyses but 5-dihydroxybenzois acid (DHB) and α-ciano-4-hydroxycinnamic acid (CHCA) are the most frequently used in lipidomics [33,42].

## 5. Lipidomics in Cancer Research

This current review gives a synopsis of lipidomic approaches in tumor characterization; this work aims to provide an overview of potential lipid biomarkers in the oncology field (Table 2). The novel lipidomic biomarkers are reviewed in an effort to underline their role in diagnosis, in prognostic characterization and in prediction of therapeutic outcomes.

### 5.1. Lung Cancer

Lung cancer represents the leading cause of cancer death for men and women. It consists of two main classes of lung tumors: about 10%–15% of lung cancers are small cell lung cancers (SCLC), while about 85% are non-small cell lung cancers (NSCLC), including squamous cell carcinoma, adenocarcinoma, and large cell carcinoma [43]. Among NSCLC, more than a third of adenocarcinoma reveals activating mutation in the KRAS gene, and overexpression in MYC [44]. The oncogenic overexpression of MYC leads to uncontrolled cell proliferation, while MYC inhibition leads to tumor regression and differentiation of cells, in preclinical models [45]. Moving from this background, Hall et al. recently applied the lipidomic profiling approach on transgenic mouse model of KRAS-driven lung adenocarcinoma with reversible activation of MYC. Using MALDI-IMS they studied the changes in lipid composition in order to characterize the lipid profile following tumorigenesis in lung tumors with high MYC activity, and subsequent deactivation of MYC. In this study, the lipid signatures for healthy and tumor lung tissue in mice with increased MYC activity were evaluated: tumor tissues presented increased signalling precursor PLs, such as the PIs and arachidonate-containing PLs, whereas healthy tissues were predominantly characterized by pulmonary surfactant lipids. PC 32:0, PC 32:1 and PGs increased in normal mouse lung tissue in comparison with tumor tissue. This result could be related to the breakdown of the fine alveolar structure with tumor progression. Lung tumors with high MYC activity showed increase in free arachidonic acid, which is released from membrane phospholipids by cytosolic phospholipase A2 (cPLA2); moreover, cPLA2 activity was increased. MYC overexpression allowed for an increase in arachidonic acid-derived eicosanoids via the lipoxygenase (LOX) and cycloxygenase (COX) pathways. In this study, after MYC deactivation, there were decreases in both cPLA2 activity and specific metabolites as well as the mRNAs associated with COX and 5-LOX. Reduction in tumor proliferation and increase in apoptosis was observed after inhibiting the COX and 5-LOX pathways in high MYC mice, highlighting these pathways as potential drug targets for lung adenocarcinoma [46]. In order to evaluate lipid profiles with diagnostic power in NSCLC, Lee et al. (2012) analyzed 21 pairs of NSCLCs and adjacent healthy tissue samples with histology-directed MALDI-IMS. Results highlighted differential lipid profiles between tumor and adjacent normal tissue samples, with PC species PC 34:1, PC 36:2, and PC 36:3 significantly elevated in NSCLC tissues. The lipid profiles resulted in different squamous cell carcinomas compared to adenocarcinomas. In an independent validation cohort, this differential lipidomic signature correctly classified the histology of 80.4% of NSCLC surgical tissue samples (41 out of 51). PC 32:0 [M + Na]^+^ (*m*/*z* 756.68) and ST-OH 42:1 [M − H]^−^ (*m*/*z* 906.89) were overexpressed in adenocarcinomas. The authors concluded that MALDI-TOF-MS analysis on lipids could assist with the histopathologic diagnosis of NSCLC, in terms of distinguishing the tumor from adjacent normal tissue and classifying the histologic type [14]. Using MALDI-MS, Pirman et al. (2013) evaluated the difference in lipid metabolism between two human NSCLC cell lines (A549 and H596), in an effort to reveal differences in the cellular metabolism of the fish-oil-derived poly-unsaturated fatty acid (PUFA), eicosapentaenoic acid (EPA), which could contribute to their differential response to EPA treatment. Previous evidence showed that the prostaglandin E3 (PGE3), derived from COX-2 metabolism of the omega-3 FA eicosapentaenoic acid (EPA), inhibited the proliferation of lung, colon and pancreatic cancer cells. The role and the metabolism of PGE3 in these cancer cells is already debated. In this study, MALDI-MS analysis showed that H596 cells incorporated four-fold higher levels of EPA into PLs than the A549 cells. Authors concluded that the lower expression of cytosolic phospholipase A2 (cPLA2) in H596 cells than in A549 cells could explain the lower synthesis of PGE3 in H596 cells in respect to A549 cells, although the expression of COX-2 resulted similarly in the two cell lines. Even though both cell lines showed similar expression of COX-2, these data supported a potential molecular cause of the H596 cells’ lower sensitivity to EPA treatment than that of A549 cells. Thus, in this study, the MALDI-MS method allowed identification of the biomarkers involved in the metabolism of omega-3 FA and to predict the effectiveness of EPA in NSCLC treatment [47]. With the aim to derive prognostic information, changes in the levels of some serum metabolites were also investigated for stratifying the different lung tumors. Guo et al. analyzed serum metabolites of patients affected by lung cancer (*n* = 58) in comparison with a healthy control group (*n* = 495). In this study, a direct-infusion positive-ion ESI Fourier transform ion cyclotron resonance (FTICR) MS was employed. In patients affected by lung tumor, 141 of the 212 serum metabolites were significantly changed when compared with the control group. In particular, this differential metabolite distribution mainly concerned PL species, such as SM 16:0/1, LPC 18:1, LPC 20:4, LPC 20:3, and LPC 22:6; these lipids were associated with lung cancer progression. Authors concluded that serum LPCs could be applied to lung cancer to differentiate patients and healthy subjects and to detect tumors early [48]. Several other pieces of evidence supported the hypothesis that altered lipid compositions can differentiate normal epithelium from tumor. Among these, Marien et al. compared GPL profiles between NSCLC tissues samples and normal tissue samples, with the MS-based phospholipidomic approach. They noted consistent differences between GPL profiles of tumor tissues and normal tissues. PI 38:3, PI 40:3, and PI 38:2 were higher in tumor samples, in comparison with the normal tissues. Conversely, SM species, like SM 40:1, SM 42:1, and SM 36:1, decreased in tumor samples compared with normal ones [49].

### 5.2. Breast Cancer

Globally, breast cancer is the most frequently diagnosed neoplasm and the first cause of cancer-related death in women [50]. Evidence has demonstrated that enzymes related to lipid synthesis are overexpressed in breast cancer tissues, with a close relationship to tumoral progression [51]. Therefore, in recent years, lipidomic analysis has been performed in order to investigate new potential biomarkers with diagnostic power. Using MALDI-IMS analysis of lipids and proteins, Kang et al. analyzed 34 pairs of surgical breast tissues (34 breast tumors with 34 adjacent normal samples) with the aim of differentiating between tumors and normal tissues (diagnostic power) and correctly classifying different subtypes of breast cancer (prognostic power). By the lipidomic analyses, PC 34:1 was shown to be overexpressed in breast cancer; moreover, the lipid MALDI MS profiles were significantly differential between the luminal, HER2+, and triple-negative subtypes of breast cancers, with important prognostic relevance [52]. Min et al. performed the analysis on four different categories of PLs (PS, PI, PG, and PA) from urine in patients with breast cancer versus healthy controls, using nanoflow LC-ESI-MS-MS. In the breast cancer group, two PS molecules (18:1/18:1 and 18:2/18:0) showed a significant increase; after surgery, their concentrations were reduced to normal levels. Total amounts of many PLs increased in cancer patients when compared to healthy controls, whereas PI 18:0/20:4 was significantly decreased in breast cancer samples. This study suggested that the lipid composition found in the urine of breast cancer patients could be used for early diagnosis [53]. Using an imaging approach able to detail the spatial distribution of PIs, Kawashima et al. performed a high resolution MALDI IMS on nine breast tumor samples and one normal breast tissue. Results showed that several PIs were specifically localized into cancer cell clusters, with a heterogeneous distribution, which identified two different populations of cancer cells: the first predominantly expressed PI 18:0/18:1, the second PI 18:0/20:3. Moreover, the latter population was associated with tumor invasion [54]. 

A quantitative approach for the lipidomic characterization of breast cancer tissues compared with surrounding normal tissues using HILIC-HPLC/ESI-MS was performed by Cifkova et al. [55]. In this study, the lipid class quantitation showed differences in lipidome of tumor and normal tissues: levels of PI, PE, PC, SM and LPC were significantly higher in tumor tissues than in healthy controls; PLs with the general formula C34:1 (mainly combination of C16:0 and C18:1) led to the association with tumor tissues for several lipid classes.

Several studies investigated the potential prognostic value of lipidomic profile in breast cancer. Hilvo et al. conducted a comprehensive lipidomic analysis by using UHPLC-MS in 267 human breast tissues [56]. They observed that the most salient difference in the lipid profiles is represented by an upregulation of PLs (PC, PE, and PI and SMs) in the negative prognostic factors tumors, such as estrogen receptor (ER)-negative tumors. In this study, (ER)-negative and Grade 3 tumors showed the high rate of palmitate de novo synthesis and its incorporation into membrane PLs, providing a prognostic signature.

Wang et al. applied MALDI IMS for describing the differential lipidomic profile between high and poor invasive breast cancer cell lines [57]. They demonstrated that in highly invasive cell types, eight lipids including SM were downregulated while 31 lipids, including PG and PA, were upregulated. This work concluded that products of FA synthesis lying into the cell membranes, such as oleic-acid-containing PG, could be an important mitochondrial failure factor involved in invasion of breast cancer cells. Doria et al. after a previous study on mouse normal breast cells and breast cancer ones, applied TLC MS on human normal breast cells and breast cancer cell lines (MCF10A, T47-D, and MDA-MB-231), in an effort to describe alterations of PL profiles between cancer and normal cell lines driving the progression of carcinogenesis. Non-malignant cells showed the highest differences in PE content, relative to total amount of PLs, whereas PA presented highest relative abundance in metastatic cells. In addition, higher levels of PCs and PI 22:5/18:0 were found in migratory cells, with metastatic ability [58]. Recently, Kim et al. performed a comprehensive and comparative metabolomic and lipidomic analysis of breast cancer cell lines with different degrees of invasiveness (MCF-7 and MDA-MB-231 cells) using GC-MS and direct infusion MS. They found that PS 18:0/20:4, PI 18:0/20:4, and PC 18:0/20:4 were markedly higher in the highly metastatic MDA-MB-231 cells than in slightly metastatic MCF-7 cells, (all *p-*values < 0.001). In contrast, the levels of PE 18:1/18:1 and PI 18:0/18:1 were markedly lower in cells with high metastatic potential than in slightly metastatic ones [59]. The partial-least-squares regression model was developed and validated for predicting the metastatic potential of breast cancer cells. A multimodal imaging approach was employed by Chughtai et al. for investigating the effects of hypoxia and necrosis on the heterogeneous lipid composition in breast tumor model [60]. By using the MALDI-IMS approach, the lipid distributions into the tumor tissue were characterized by spatial heterogeneity: in particular PC 16:0/16:0, PC 16:0/18:1, PC 18:1/18:1 and PC 18:0/18:1 were localized in accessible tumor areas, whereas LPC 16:0/0:0 were localized in necrotic tumor regions. The MALDI-IMS images revealed that palmitoylcarnitine, stearoylcarnitine, PC 16:0/22:1, and SM d18:1/16:0 are predominantly localized in the hypoxic tumor regions [60]. De novo lipogenesis increased most solid tumors early on, potentially affecting the chemo-sensitivity of cancer cells. In an effort to investigate the potential predictive value of changes in lipidome, Hilvo et al. explored if the lipidomic profiles are associated with pathologic complete response (pCR) in breast cancer patients, receiving neoadjuvant chemotherapy [61]. This study highlighted that serum triacylglycerol (TGs) containing C18:1 fatty acyl chains were at lower concentrations in patients with pCR. Previously, Wei et al. performed a serum metabolite profiling in order to identify a potential biomarker in response to neoadjuvant chemotherapy for breast cancer (predictive power). They applied LC-MS in combination with NMR spectroscopy data, in a population of breast cancer patients having undergone complete (*n* = 8) or partial (*n* = 14) chemotherapy or having had no response (*n* = 6) to chemotherapy; the serum metabolic profiles of patients with pCR were significantly different in threonine, glutamine and isoleucine levels; moreover, the free linoleic acid (C18:2) was detected in lower levels in pCR patients [62].

### 5.3. Prostate Cancer

Prostate cancer is the most common malignancy in males, and the second-leading cause of cancer-related death in men in Western countries [63]. Both screening and early diagnosis represent the main issues in the care of prostate cancer. Moreover, determination of a therapeutic strategy based on outcome prediction is needed. New biomarkers have a promising role in leading these clinical needs, since none of the currently used molecules in prostate cancer, including PSA, is satisfactory. The potential diagnostic role of lipidomic approaches in prostate cancer setting was evaluated in several studies. Using nLC/ESI-MS/MS, Min et al. [64] compared the PL concentrations in urine samples between healthy volunteers and patients with prostate cancer. They found that PS 18:0/18:1, PS 16:0/22:6 were significantly increased in patient samples, while PS 18:1/18:0, PS 18:0/20:5, had significantly decreased in comparison with healthy controls. By using immunohistochemistry (IHC) on tissue microarray (TMA) slides, Zhou X. et al. investigated if the expression of lysophosphatidylcholine transferase 1 (LPCAT1) was correlated with the cancer progression in order to elucidate a possible diagnostic and prognostic role of LPCAT1 in prostate tumors. It was already known that LPCAT1 plays a key role in remodelling PLs, and were overexpressed in several carcinomas (colorectal and prostate) as compared to normal mucosa [65,66]. The study showed a significant difference in LPCAT1 IHC mean scores among benign, malignant and metastatic prostate cancer groups. The metastatic prostate cancer group revealed the highest LPCAT1 IHC mean score. Moreover, stratifying according to the Gleason score into three subgroups (low grade, intermediate and high grade, which are directly correlated with a worse prognosis) they observed a significant difference in LPCAT1 IHC mean score, with the high grade group (Gleason score > 7) showing significantly higher levels than that in the other subgroups. Furthermore, LPCAT1 IHC mean score was significantly higher in non-organ-confined prostate cancer in contrast to organ-confined prostate cancer (*p* < 0.0001). The LPCAT1 expression level could differentiate between prostate cancer subtypes, and could correlate with the Gleason score and tumor staging systems, as principal prognostic index. The LPCAT1 IHC mean score was significantly higher in the patients with biochemical recurrence and/or metastasis during follow-up than those who did not (*p* = 0.00000409). These data highlighted that the LPCAT1 expression level could be both a predictive and a prognostic index of occurring biochemical recurrence and/or metastasis [67]. Goto and co-workers performed HR-MALDI-IMS in negative mode technique on human prostate tissue samples obtained from patients with prostate cancer at the time of radical prostatectomy, in an effort to compare lipidomic signatures between tumor and benign epithelium. The HR-MALDI-IMS was employed on 38 human prostate tissue samples (14 in discovery set and 24 in the validation set): the expression of PI 18:0/18:1, PI 18:0/20:3 and PI 18:0/20:2 was significantly higher in cancer tissue than in benign epithelium. The authors proposed that the differences in PI expression levels could be correlated with the activity of the PI3K signalling pathway [68]. Afterwards, Goto et al. (2015) evaluated the lipidic signature on 31 tissue samples from prostate cancer patients by high-resolution (HR) MALDI-IMS. Results showed five lipid species significantly lower in cancer than in benign tissues, specifically: LPC 16:0/OH + H^+^, LPC 16:0/OH + Na^+^, LPC 16:0/OH + K^+^, LPC 16:0/OH + matrix + H^+^, and SM d18:1/16:0 + H^+^. A low level of LPC 16:0/OH in cancer tissue was associated with biochemical recurrence after radical prostatectomy [69]. Using ESI-MS/MS lipid profiling, Patel et al. analyzed distribution and concentrations of serum PLs from newly diagnosed prostate cancer patients and healthy subjects. Interesting species of cholesterol (CE), dihydrosphingomyelin (DSM), PC, eggPC and eggPE were associated with prostate cancer. The cut-off values derived in the tested serum (ePC 38:5 > 0.015 nmoles, PC 40:3 < 0.001 nmoles and PC 42:4 < 0.0001 nmoles) were associated with a predictability of 94% for the absence of prostate cancer; whereas for the cut-off value of ePC 38:5 < 0.015 nmoles, PC 40:3 > 0.001 nmoles, and PC 42:4 > 0.0001 nmoles, the predictability of prostate cancer disease was high. The authors concluded that a combination of serum ePC 38:5, PC 40:3 and PC 42:4 could be predictive for detecting prostate cancer, if confirmed in a larger dataset [70]. Duscharla and co-workers recently employed ESI-MS/MS and GC-MS in an effort to identify significantly altered lipids in serum of prostate cancer patients. With the aim to provide a specific lipidomic signature they investigated complete lipid profiles including different classes of lipids: FAs, TGs, DGs and PLs. This lipidomic approach revealed 24 lipids significantly different in serum of cancer patients (*n* = 18) compared to normal ones (*n* = 18). PC (39:6) and FA (22:3) classified samples with higher certainty, cataloging patients with 100% sensitivity (all 18 control samples are classified correctly) and 77.7% specificity, with *p*-value of 1.612 × 10^−6^ in Fischer’s exact test, showing good sensitivity, specificity and accuracy in detection of prostate cancer. Thus, they proposed PC 39:6 and FA 22:3 as possible serum biomarkers in prostate tumors, even if a further validation is needed [71].

### 5.4. Colorectal Cancer

Colorectal cancer is the fourth most common cancer in men and women, and is the second leading cause of mortality in the United States [50]. Research of sensitive, reliable, and specific biomarkers is a main issue in oncology. To determine if LysoPLs could be used as markers in colorectal tumors, Zhao Z. et al. analyzed plasma LPCs from 133 colorectal cancer patients and 125 healthy controls using LC-MS. Results showed plasma levels of different LPC forms, including 18:1 and 18:2, were significantly reduced in colorectal cancer patients, suggesting that these lipids could represent potential diagnostic biomarkers. The multivariate analysis in the validation set found a specificity of 93% and a sensitivity of 82% in revealing cancer patients in contrast to healthy controls. They proposed a predictive model able to properly classify eight (89%) of nine T1 stage tumors, suggesting that it represents a sensitive marker of early-stage colorectal cancer detection [72]. Dobrzyńska et al. focused on changes of PL content (PI, PS, PE, PC) in cell membranes of colorectal cancer of pT3 stage. By using HPLC, qualitative and quantitative composition of PLs in the membrane were determined on adenocarcinoma tissue samples obtained from 18 patients. They concluded that cancer transformation was accompanied by an increase in total concentration of PLs; moreover, metastatic tumor cells were characterized by a higher PC/PE ratio than malignant neoplasm cells without metastases [73]. Since these changes in membrane PL levels can influence cell proliferation, motility and tumor progression, Kurabe and co-workers applied mass microscopy to human colorectal cancer tissues as a non-targeted screening for PC biomarkers for obtaining a lipid profile. With PCA, they identified a difference between tumoral cells and adjacent healthy colorectal mucosal cells. They found one PC 16:0/16:1 as a differentially expressed lipid between the neoplastic and non-neoplastic mucosa samples. In an in vitro analysis, we showed that LPCAT4 is involved in the deregulation of PC16:0/16:1 in colorectal cancer. The IHC analysis showed overexpression of LPCAT4, which is implicated in the increase of PC 16:0/16:1. These data indicated the potential role of PC 16:0/16:1 for the clinical diagnosis of colorectal cancer [74]. Mirnezami R. et al. applied MALDI-IMS on colorectal cancer tissue and adjacent healthy mucosa obtained from 12 patients undergoing surgery. This study showed elevated levels of PC 16:0/18:1, LPC 16:0 and LPC 18:1 in tumor samples. Moreover, they demonstrated different tissue regions in the same colorectal cancer microenvironment in terms of distinct lipid characteristics, presenting the evidence of a variety of cancer sub-types [75]. Analyzing serum metabolites from colorectal cancer patients (*n* = 101) and healthy controls (*n* = 102) by GC-TOFMS and UPLC-Q TOF-MS, Tan B. et al. showed a specific metabolic signature in colorectal cancer samples. The proposed metabolomic model correctly classified the validation set of 39 colorectal cancer patients and 40 healthy controls. The authors concluded that the panel of serum markers provided great potential as a non-invasive diagnostic method for the detection of colorectal tumors [76]. In the metabolomic research setting, Thomas et al. proposed a lipid signature in human liver metastasis from colorectal cancer, by using a histology-driven IMS approach: they found lipid species significantly up- and down-regulated in the tumor region. In particular, the MALDI-IMS images of PE species, PE 38:6, PE 40:4 were upregulated in cancer tissues [77]. Coviello et al. aimed to analyze the FA profile in red blood cell membranes of 13 patients affected by colorectal cancer and 13 healthy controls. Using GC, FAs were extracted from erythrocytes’ membranes and quantified. Results evidenced that colorectal samples had significantly lower percentage of n-3 PUFAs in comparison to controls (5.1% vs. 8.0%, respectively). Moreover, in cancer patients, a higher ratio n-6-PUFA/n-3-PUFA was observed [78].

### 5.5. Ovarian Cancer

Epithelial ovarian cancer is a gynecologic neoplasm, the fifth most common cause of cancer mortality in women. Since less than 40% of women with this cancer can be cured, identification of a biomarker is important to improve diagnoses and therapies as well as reduce mortality. Zhang et al. applied the UPLC-ESI-QTOF-MS to probe potential diagnostic biomarkers in the plasma. They demonstrated that plasma LysoPA (LPA) had increased levels in patients with ovarian cancer. They found as important biomarkers LPCs, PCs and GLs. The first lipid species were increased, the other two decreased in patients with ovarian cancer compared to healthy women. Authors suggested that deregulation of phospholipase A2 (PLA2) activity could produce a high level of LPC, providing a potential marker and therapeutic target [79]. Kang et al. analyzed lipid and protein profiles in ovarian cancer tissues with histology-directed MALDI MS. They compared metabolomic profiles of 23 samples of ovarian cancer to six adjacent normal ones. Levels of PC 32:3, 34:1 and 36:2 were higher in ovarian cancers. The authors concluded that modifications in lipid profiles could differentiate ovarian cancer from adjacent normal tissue [80]. The prognostic value of lipidome changes in ovarian neoplasm was investigated by several studies. Zhao et al. performed lipidomic analyses using UHPLC-ESI MS/MS. They generated highly aggressive ovarian cancer cell lines (ID8-P1 lines) from less aggressive ID8-P0 (without in vivo passage) in mice, and they examined lipidomic analyses in these two different cell groups. Several classes of lipids were differentially present in ID8-P1 versus ID8-P0 cells, with a notable increase in TG levels under detachment stress in cells. Since epithelial ovarian cancer cell detachment represents an important feature of treatment failure, authors concluded that TG could be a future study issue in leading new treatment modalities [81]. Using ESI-MS, Sutphen et al. evaluated the role of LPA and other LPLs as markers for diagnosis and/or prognosis of ovarian cancer. This study reported statistically significant differences in LPL levels between preoperative plasma samples (*n* = 45) of ovarian cancer patients and those of healthy controls (*n* = 27). In patients with early-stage disease, the statistically significant elevations in LPL levels was reported, supporting the utility of LPL, especially LPA 16:0 and LPA 20:4, as possible biomarkers for early detection of ovarian tumors [82]. Meleh et al. performed HPLC with ESI-MS/MS in order to evaluate LPA species in serum of healthy controls; there were 55 women (aged 20–65 years) and patients with benign (*n* = 65) and malignant (*n* = 50) ovarian neoplasms. This approach allowed the extraction of the LPA species from the sera in one phase, using a methanol–chloroform mixture. Healthy controls’ sera showed significantly lower LPA levels in comparison with those of the ovarian cancer patients, while no differences were evident between malignant or benign ovarian cancers [83]. Thanks to their higher levels, in very early ovarian cancer stages, LPA could be used as a diagnostic biomarker, even if they cannot distinguish between malignant and benign tumors. Increased levels of LPA in biological samples of patients with ovarian cancer compared to healthy individuals were also reported in several studies, supporting LPA as a potential biomarker for detection of ovarian tumors [84,85,86]. After reporting that LPA represented a potential biomarker for ovarian and other gynecologic cancers, Xiao and colleagues developed an ESI-MS-based method to analyze LPA aiming to increase accuracy, sensitivity and specificity of the test. They studied potential diagnostic and/or prognostic biomarkers between LPA species, LysoPI (LPI), LysoPS (LPS), and LPC in patients with ovarian and other gynecologic cancers, in respect to healthy people. The ovarian cancer patients presented both LPA and related LPLs, in particular LPA 16:0, LPA 18:0, 18:1, 18:2, LPI 16:0, LPI 18:0, LPI 20:4 [85]. Sedláková et al. analyzed the plasma LPA levels of 133 patients (including 60 patients with ovarian cancer, 43 women without ovarian diseases and 30 patients with benign diseases), using capillary electrophoresis with indirect ultraviolet detection. A significantly higher plasma LPA level was found in patients with ovarian cancer, compared with controls and patients with benign ovarian tumor (*p* < 0.001). In this analysis, the plasma LPA levels were correlated with the ovarian staging system (according to the International Federation of Gynecology and Obstetrics—FIGO—stages) and histological type [87]. This finding could sustain the diagnostic and prognostic value of LPA in ovarian neoplasms. In a further update, the same authors compared plasma LPA levels in ovarian cancer patients (*n* = 81) in women with benign ovarian tumors (*n* = 51) and in women with no ovarian pathology (*n* = 27) during a five-year period. Using capillary electrophoresis with indirect ultraviolet detection, results confirmed the significantly higher plasma LPA level in the ovarian cancer group (*p* < 0.001). Even though plasma LPA levels were associated with the FIGO classification, the histological subtype and grading of ovarian cancer did not correlate to the plasmatic LPA levels in this cohort. Thus, LPA could represent a potential diagnostic biomarker in ovarian cancer, which would be particularly useful for early detection [88]. Zaho et al. applied HPLC/ESI-MS to perform a PL metabolic profiling in ovarian tumors including plasmalogen PE, PC, plasmalogen PC, SM and LPC resulting in the differential phospholipids among malignant, benign and normal groups (*p* < 0.05) [89].

### 5.6. Pancreatic Cancer

Pancreatic cancer is the fourth most common cause of cancer-related death [50]. Considering the high frequency of mortality in patients affected by this disease and the high discovery of pancreatic neoplasm when the pathology is already metastatic, new studies for identifying characteristics that could help in early diagnosis and cure are necessary. Transcriptomic and proteomic analyses have already been utilized to study pancreatic cancer, so lipidomic analyses represent a further contribution to finding new potential diagnostic, prognostic and predictive biomarkers. Since SMs, ceramide species and their metabolites regulate growth, migration and metastasis of pancreatic neoplasms [90], Jiang et al. utilized an MS-based lipidomic approach to evaluate changes in the SMs in pancreatic cancer tissues and plasma specimens. Studying the tissues, the authors found that patients with lymph node metastasis N+ showed higher levels of ceramides C16:0 and C24:1 compared to patients without lymph node metastasis or patients affected by pancreatitis. Moreover, pancreatic cancer patients with nodal disease showed elevated levels of ceramide metabolites, including phosphorylated (sphingosine- and sphinganine-1-phosphate) and glycosylated (cerebroside) species. In regards to pancreatic tissue, cerebrosides consisting of a C18:0, C20:0, C22:0, C24:0, or C24:1 FA demonstrated low levels regardless of metastasis status. Contrary to the tissue, the plasmatic cerebroside species (C16:0, C20:0, C22:0, C24:0, C24:1) were significantly elevated in N+ patients. They concluded that the SL metabolism was changed in tissue and plasma of pancreatic cancer patients: an aggressive or metastatic disease is characterized by alterations in SL metabolism that could represent a diagnostic and prognostic indicator in risk-stratified patients [91]. Since several studies have demonstrated that LPA is a crucial component of ascites which could lead to metastases in pancreatic neoplasm, Liao and colleagues considered LPA1-3 in LPA-induced activation of FAK (focal adhesion kinase) and paxillin (a focal adhesion protein). LPA is involved in cell proliferation, survival, angiogenesis and cell migration; moreover, it can be implicated in invasion and metastasis of pancreatic cancer [92]. LPA1/2 and 3 receptors are overexpressed in cancer cells and implicated in their motility [93]. The possible involvement of LPA1-3 specific inhibitor, called Ki16425, was debated. The authors found that the suppression of LPA1-3 determined the inactivation of FAK and paxillin and repressed cell migratory ability; moreover, the effect of LPA-stimulated migration was suppressed by inhibition of LPA-1 activity. Therefore, these findings support the prognostic role of LPA, and could promote novel predictive targets for treatment of pancreatic neoplasm [94]. To probe potential diagnostic biomarkers, Macasek et al. analyzed FA profile in plasma of patients affected by pancreatic cancer, in accordance with tumor staging and patient survival. Using GC, the authors analyzed 84 patients affected by pancreatic neoplasm vs. 68 healthy controls. They found an increment of monounsaturated FA (MUFA) in pancreatic cancer patients; in particular, the authors found that patients showed an increment in de novo synthesis of FA with transformation into MUFA [95]. Zuijdgeest-van Leeuwen et al. investigated whether plasma n-3 FA concentrations were reduced in patients affected by pancreatic, lung and esophageal cancer in comparison to healthy controls. The deficiency of n-3 FA is generally correlated to intestinal malabsorption, decreased intake and inflammatory condition. In this study, pancreatic cancer patients showed reductions in n-3 fatty acid concentrations in plasma PLs and cholesteryl esters (CEs). The authors concluded that there were differences in plasma FA compositions between the different tumor types [96].

### 5.7. Gastric Cancer

Gastric cancer is the third leading cause of death from cancer worldwide [1]. Gastric cancer is often diagnosed in an advanced stage and in some parts of the world, it is a major challenge for healthcare professionals. Several lipidomic studies were conducted to find lipid signature with diagnostic or prognostic significance in gastric cancer.

In order to differentiate lipid alterations related to the development of gastric cancer, Uehara and colleagues compared the lipid content of gastric cancer tissue and adjacent non-neoplastic mucosa from 12 samples, using the MALDI-IMS technique. The authors focused on the signal at *m*/*z* 798.5, representing PC 16:0/18:1, that was notably higher in the cancerous lesions than in the adjacent healthy mucosa. The intensity of the signal at *m*/*z* 496.3, an LPC 16:0, was significantly lower in cancer lesions, predominantly in differentiated adenocarcinomas. Most of the differences between cancer lesions and non-neoplastic mucosa corresponded to PLs: the majority of were PC and LPC. These changes in lipid content could compromise membrane fluidity and signal transduction in cancer cells, affecting tumorigenesis and gastric cancer progression. Levels of LPCAT1 were higher in gastric cancer tissue than in adjacent non-neoplastic mucosa, and this overexpression was predominant in differentiated adenocarcinomas, so LPCAT1 could play important roles in the tumorigenesis of differentiated adenocarcinomas compared to undifferentiated ones. Moreover, LPCAT1 expression levels were positively correlated with tumor differentiation and negatively with lymph node metastasis, tumor stage and tumor depth, assuming a potential prognostic value [97]. Using two gastric cancer cell lines, Shida et al. investigated whether sphingosine 1-phosphate (S1P) induces phosphorylation of two receptors tyrosine kinases (RTKs), such as epidermal growth factor-1 receptor (EGFR) and c-Met, whose signals are crucial in gastric cancer progression. They found that only S1P, but not sphingomyelin and sphingosine, induced transactivation of c-Met and EGFR, so they could conclude that the S1P receptor mediates both transactivations. Considering that RTKs could regulate different mechanisms of proliferation, differentiation, motility and survival, their dysregulation could be implicated in the development and progression of numerous human cancers, particularly human gastric cancer. S1P may be a potent stimulator of gastric cancer progression thanks to the activation of various RTK signaling pathways. Therefore, S1P could be considered a prognostic index of tumor progression and could be used as a target of new molecular therapies (antagonists of S1P), as a predictive factor of response [98].

### 5.8. Bladder Cancer

Bladder cancer is the sixth most common cancer, representingmore than 90% of all urothelial tumors. Urothelial (transitional cell) carcinomas can originate from the renal pelvis to the ureter, bladder, and proximal urethra, where the transitional epithelium lined the mucosa [99]. Dill et al. used the DESI-IMS method to investigate diagnostic biomarkers on 20 pairs (40 tissue samples) of human bladder cancers from adjacent normal bladder tissue samples, evaluating lipidic species. Their results showed important changes in PI, PS and FA levels. There was an increment of PS 18:0/18:1, PI 18:0/20:4 and FA 18:1 in tumor tissues in comparison to healthy ones [100]. Previously, the same authors (Dill et al., 2009) studied lipid profiles of tissue sections of canine invasive transitional cell carcinoma (TCC) of the urinary bladder compared to adjacent normal tissue from four different dogs, using DESI-MS, with the same aim of representing a diagnostic tool. Positive and negative ion modes allowed for distinguishing between disease and healthy tissue through multiple marker lipids and multiple free FAs. The results showed differences in lipid species distributions (represented by GLs, SLs and free FAs), in both negative and positive ion modes, between tumors and their adjacent normal tissues. In particular, the tumor tissue had increased absolute intensities for the signals relative to PS 18:0/18:1, PG 18:1/18:1, PI 16:0/18:1, PI 18:0/18:1, PS 18:1/18:1, PC 34:1 and PC 36:2 when compared with normal tissue. Moreover, SM 18:1/16:0 was increased in normal tissues compared to the diseased tissues [101].

### 5.9. Esophageal Carcinoma

Esophageal cancer is the sixth most common cause of cancer deaths around the world and, in the developing nations, where is most endemic, it is the fourth most common cause of cancer deaths [102]. There are two main histology subtypes: squamous cell carcinoma (SCC) and adenocarcinoma [103]. Nowadays, adenocarcinoma gradually increased in frequency both in men than in women [104]. In the aim of discovering biomarkers with both diagnostic and predictive purposes, Xu et al. studied plasma of patients affected by esophageal SCC before, during, and after chemoradiotherapy (CRT), compared to a healthy control group, using LC-MS/MS. They found lipid differences not only between patients and healthy controls but also between pre-treatment and post-treatment samples. Among LPCs, FAs, l-carnitine, acylcarnitines, organic acids, and a sterol metabolite, 18 samples were altered in cancer patients compared to healthy controls. Moreover, they observed an important decrease in cholic acid levels—the most significant metabolite of cholesterol—in esophageal cancer patients. There were 11 metabolites differentiated between pre- and post-treatment patients, including amino acids, acylcarnitines, and LPCs (in particular LPC(16:1)). Of these, octanoylcarnitine and decanoylcarnitine were strongly correlated with treatment effectiveness. These biomarkers are potentially useful in diagnosis, in monitoring therapeutic responses and predicting outcomes [105]. Liu and colleagues analyzed 53 pairs of plasma samples from SCC patients and healthy controls (17 vs. 29), using UPLC-ESI-TOFMS, with the result of finding 25 upregulated and five downregulated metabolites, for early diagnosis of SCC. PS, PA, PC, PI, PE and sphinganine 1-phosphate were overexpressed in cancer patient plasma compared to healthy controls. This demonstrated the critical role of PLs in the SCC oncogenesis; therefore, the identification of novel biomarkers could be an important strategy to reaching an early diagnosis that could improve clinical outcome [106]. Biomarkers’ diagnostic value has been recently evaluated by Uchiyama and colleagues, with the intention of distinguishing biomolecule distributions through IMS, in 11 signals between neoplasm and stromal regions. Since the border between cancer (the most frequent histology is SCC) and stromal regions is often unclear and surgery is required to resect a large part of the organ, new methods of diagnosis to make a non-invasive surgery are needed. This study identified PC 16:0/16:1 in the cancer region and PC 18:1/20:4 in the stromal region, expressed in different rates. Thus, these lipids could be used as potential biomarkers to distinguish neoplastic regions from normal regions in esophageal carcinoma [107].

### 5.10. Kidney Cancer

Renal cell carcinoma (RCC) includes several histological subtypes (clear cell, papillary, chromophobe, medullary and collecting duct and unclassified subtypes) [108], with clear cell RCC representing the most-common histotype. Since this tumor is generally asymptomatic at an early stage, high metastasis rates are reported due to late diagnosis. Thus, identification of a specific biomarker is essential to allow for early detection. Moreover, RCC is characterized by high chemo- and radio-resistance, limiting the efficacy of the few treatment options, while targeted therapies have demonstrated a survival benefit [109]. RCC metabolic signature is characterized by alterations in energy metabolism pathways, among which are FAs’ catabolism pathways, with essential implications in cell growth and proliferation [110]. Currently, diagnosis is based on imaging techniques, but early and cancer-specific biomarkers are lacking; development of new approaches for early diagnosis, prognosis and prediction could impact patient’s outcome [111].

By using an LC-MS-based method with reversed phase (RP) LC and HILIC separations, Lin et al. found serum PE (P-16:0e/0:0), ganglioside GM3 (d18:1/22:1), C17 sphinganine, and SM 18:0/16:1(9Z) able to discriminate between bladder tumors, kidney tumors and healthy controls [112]. Jones et al. aimed to differentiate normal tissue comparedt to RCC tissues obtained by nephrectomy from patients with clinical moderate risk of disease progression. In the group of patients analyzed, half of them progressed to metastatic carcinomas three years after nephrectomy, whereas the other half were disease-free for five years. There were no histopathologic differences between these two populations and there were no features which could explain or predict disease progression. MALDI-IMS profiling of protein and lipid expression was performed on those tissues, in order to identify panels of proteins and lipids able to distinguish tumor from non-tumor tissues, as well as recurrent disease progressors from non-progressors. There were 39 lipid species identified that were most discriminatory for tumor versus healthy tissue or recurrent versus non-recurrent tumor conditions [113].

Saito et al. used the lipidomic approach (LC-MS-based lipidomic platform, which combined a non-targeted approach for PLs, SLs, neutral lipids, and acylcarnitines with a targeted approach for PUFA and their metabolites) to identify lipids that were differentially present between RCC tissues and normal cortex in the same affected patients. Of 326 lipids analyzed in 49 RCC patients, PLs, CEs and TGs were higher in cancer cells, whereas PLs (no PCs) and PUFA were lower. The lower PE lipid content represented a significant diagnostic feature of RCC [114]. Using HILIC-HPLC/ESI-MS, Cifkova et al. studied the differences in 20 kidney cancer patients between polar lipids in tumors and in their surrounding healthy tissues [115]. They found that PE, LPC and SM had major differences between normal and pathological tissues, with a lower number of tumor ones. In particular, there was an upregulation of PE 36:1, PC 38:4, PC 36:2 and PC 32:0, whereas PE 34:2, PE 36:4, PE 38:4, PC 34:1, PC 34:2, PC 36:4 and PI 36:4 were downregulated in tumor tissues when compared with normal ones. To investigate the potential diagnostic value of lipidomic changes between RCC and adjacent healthy tissue, Dill et al. used DESI-MS in 20 patients (respectively nine affected by clear cell RCC and 11 with human papillary renal cell carcinoma). Partial least square discriminate analysis (PLS-DA) well distinguished tumor from normal tissue with misclassification rates obtained from the validation set of 14.3% for papillary RCC and 7.8% for clear RCC. It was also performed in order to discriminate between papillary and clear RCC with a misclassification rate of 23%, as determined from the validation set. GPLs showed a different distribution in normal and cancerous tissues: the tumor tissue revealed higher absolute intensities for the lipid species PI 18:0/20:4, PS 18:0/18:1, PG 18:1/18:1 and PI 22:4/18:0; the FA 12:0 showed an increased absolute intensity in the normal tissue, correlating inversely with the cancerous ones [116].

### 5.11. Thyroid Cancer

Thyroid carcinoma is uncommon. Likely thyroid nodules, thyroid carcinoma occurs 2 to 3 times more often in women than in men [43]. There are different histologic types that include differentiated carcinomas, with the variant of papillary (80% of all thyroid cancer), follicular and Hürthle cell types, medullary carcinoma and anaplastic carcinoma; the last one is an aggressive undifferentiated tumor. Prognosis varies depending on the histology: anaplastic carcinoma often causes death; however, most thyroid carcinoma deaths are from papillary, follicular, and Hürthle cell carcinomas [117]. Ishikawa et al. performed an IMS analysis of PLs in seven cases of thyroid papillary cancer, comparing normal tissues and neoplasm in order to identify cases of poor prognosis and to improve life expectancy, predicting anaplastic transformation before it happens. They investigated PLs in thyroid cancer and detected PL overexpression in thyroid neoplasm. They identified higher levels of PC 16:0/18:1 and PC 16:0/18:2 and SM d18:0/16:1 in thyroid papillary cancer compared to normal thyroid tissue. Despite the intensities of most *m*/*z* values in neoplasm being higher than those in normal regions, the intensity of *m*/*z* 772.5, 782.5 and 848.5 in cancer regions was lower compared to healthy regions. The *m*/*z* 798.5 peaks contained FA C18:1 that were expressed intensely in thyroid cancer regions [118]. More recently, Guo et al. investigated changes in lipids present in malignant thyroid cancer (MTC) and benign thyroid tumor (BTT) tissue and sera, through MALDI-Fourier transform ion cyclotron resonance MS and IMS. They studied 36 tissue samples consisting in 16 MTC, 5 BTT and 15 adjacent non-tumor tissues (ANT) from 23 patients and 289 serum samples from 124 MTC patients, 43 BTT patients, and 122 normal controls. Aiming to identify biomarkers able to differentiate among MTC patients, BTT patients and healthy individuals, the authors found changes in ten lipid levels: PC 34:1, PC 36:1, PC 38:6, PA 36:2, PA 36:3, PA 38:3, PA 38:4, PA 38:5, PA 40:5, and SM 34:1 in both tissues and sera. PC 34:1, PC 36:1 and PC 32:0 were overexpressed in MTC tissues; in BTT tissues, as compared to MTC and ANT tissues, there was an increment of PC 36:1 and a decrement of SM 34:1. Serum level of PA 36:62, PA 36:3, PA 38:4, PA 38:5, and PA 40:5 decreased in BTT and MTC compared with normal. A biomarker of PC 34:1 exhibited excellent diagnostic ability in differentiating both MTC and BTT patients from normal individuals; a panel which included PA(36:3) and SM(34:1) could distinguish between MTC and BTT; and a panel consisting of PC(34:1), PA(36:3), and SM(34:1) could differentiate MTC patients from both BTT patients and normal individuals. In conclusion, biomarkers can provide relevant diagnostic ability for differentiating between MTC, BTT patients and normal individuals, and they can be a powerful tool for faster screening and diagnoses [119].

## 6. Lipidomic Highlights in Cancer Research

This review widely summarizes the latest results obtained by lipidomics in cancer investigations, highlighting a deregulation of several lipid species in cell lines, tissues, plasma, serum, and/or urine. These pieces of evidence suggest that a typical phospholipid (PL) composition showing as different glycerophospholipids (GPLs), sphingolipids (SLs), fatty acids or acyls (FAs), and sterol lipids (STLs) may represent a diagnostic tool in early detection of most cancer types.

This diagnostic power was widely documented for GPLs, such as phosphatidylcholines (PC), phosphatidylethanolamine (PE), phosphatidylinositol (PI), lysophosphatidylcholine (LPC), and for SL species, since they are very abundant and easy to study through mass spectrometry (MS). Particularly in lung and breast cancer studies, it seems that there are higher levels of GPLs and SLs than in healthy controls.

Moreover, the GPL levels were correlated with tumor-negative prognostic factors, suggesting a potential role in tumor progression.

Among studied tumors, ovarian cancer appears to be particularly related to high levels of lysophosphatidic acid (LPA) and LysoPI (LPI), as reported by several aforementioned studies. Therefore, we may conclude that these lipids could represent diagnostic biomarkers of ovarian malignancies. All this evidence, obtained from peripheral biofluids, such as serum and plasma, is well aligned with the literature since the bioactive LPA stimulates cell proliferation and migration [120].

Another important evidence emerges from a lipidomic study of pancreatic tumors. In this tumor type, results suggested an imbalance of the pathway of ceramides characterizing tumor tissues and plasma of patients compared with healthy controls. Also this biomedical result falls well within the scientific literature, as ceramide is a powerful tumor suppressor, potentiating signaling events that drive apoptosis, autophagy responses and cell cycle arrest [121].

The last consideration of this review concerns the levels of lipids in biofluids investigation. For example, phosphatidyloserines (PSs) were found differentially expressed in urines of both breast [53] and prostate [64] cancer patients. Serum triacylglycerols (TGs) and linolenic acid seem to have predictive value of therapy response in cases of breast cancer patients receiving neoadjuvant chemotherapy [61,62], whereas plasma levels of different LPCs in colorectal cancer [72] or in prostate cancer [69] were also described. These studies provide a cancer lipid fingerprint in different accessible biofluids, thereby opening the way for the discovery of diagnostic biomarkers able to properly classify cancer patients. Therefore, lipidomic approaches could find additional biomarkers with high sensitivity and specificity, often obtained with minimal invasiveness. Significantly, standard clinical-pathological features are as of yet unable to predict the clinical outcome of most tumor types, thus supporting the need to obtain new biomarkers.

## Figures and Tables

**Table 1:** Classification of lipids based on structures. The table lists different lipid classes and the main subclasses cited in the text. FAs: Fatty acids; GLs: Glycerolipids; GPLs: Glycerophospholipids; SLs: Sphingolipids; STs: Sterol lipids; PRLs: Prenol lipids; SCLs: Saccharolipids; R1: Aliphatic chain; R2: Mono-unsaturated chain; R3: Poly-unsaturated chain; R4: Polar head of PCs; R5: Polar head of PEs; R6: Polar head of PGs; R7: Polar head of PSs; R8: Polar head of PIs; R9: Structure of Pas; R10: Structure of Ceramide; R11: Polar head of SMs.

Class of Lipids	Subclasses of Lipids	Structures
FAs	Saturated FAs, Mono-Unsaturated FAs, Poly-Unsaturated FAs (Arachidonic acid and derivatives), Hydroxy Fas	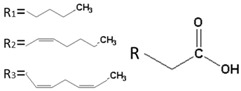
GLs	Monoacylglycerols, Diacylglycerols, Triacylglicerols (TGs)	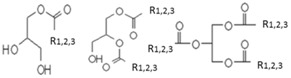
GPLs	PC, PE, PG, PS, PI, PA, LPLs, Plasmalogens (ether phospholipids)	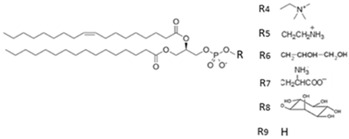
SLs	SM, Lyso SM, Ceramides, Cerebrosides, Gangliosides, Sulfatides	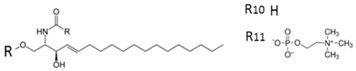
STLs	Sterols, Steroids, Steroid conjugates	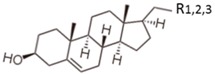
PRLs	Isoprenoids, Quinones and Hidroquinones, Polyprenols	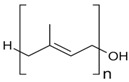
SCLs	Acylaminosugars, Acylaminosugars glycan, Acyltrehaloses	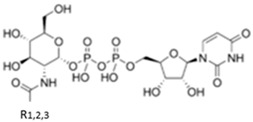

**Table 2:** The table lists the main lipids studied in this review in various tumor types, specifying with a symbol “X” if these have been found to be up or down-regulated in the respective sample type, as well as if they have diagnostic, prognostic or therapy response predictive value X*: an analysis of spatial distribution was performed; X°: lipids have predictive value of therapy response in case of patients receiving neoadjuvant chemotherapy; X§: study reveals a specific cut-off of the lipids with a diagnostic power; NS = Tumor type not specified. Ref: References; GPLs: Glycerophospholipids; FAs: Fatty Acids; SLs: Sphingolipids.

Lipid Class	Name of Lipid	Tumor Type	Sample Type	Up	Down	Diag. Factor	Prognostic Factor	Predictive Factor	Reference
GPLs	PI arachidonate-containing phospholipids	Lung cancer (adenocarcinomas)	Tissue	X	–	X	–	–	[46]
GPLs	PC 32:0	Lung cancer (adenocarcinomas)	Tissue	–	X	X	–	–	[46]
	PC 32:1								
	PGs								
FAs	free arachidonic acid	Lung cancer (adenocarcinomas)	Tissue	X	–	X	–	–	[46]
GPLs	PC 34:1	Lung cancer (NSCLC)	Tissue	X*	–	X	–	–	[14]
	PC 36:2								
	PC 36:3								
GPLs, SLs	PC 32:0	Lung cancer (NSCLC)	Tissue	X*	–	X	–	–	[14]
	ST-OH 42:1								
	*m*/*z* 906.89								
FAs	EPA	Lung cancer (NSCLC)	Tissue	X	X	–	–	X	[47]
GPLs	SM 16:0/1	Lung cancer (Different cancer type)	Serum	X	–	X	–	–	[48]
	LPC 18:1								
	LPC 20:4								
	LPC 20:3								
	LPC 22:6								
GPLs	PI 38:3	Lung cancer (NSCLC)	Tissue	X	–	X	–	–	[49]
	PI 40:3								
	PI 38:2								
SLs	SM 40:1	Lung cancer (NSCLC)	Tissue	–	X	X	–	–	[49]
	SM 42:1								
	SM 36:1								
GPLs, SLs	PC	Breast cancer NS	Tissue	X	–	–	X	–	[56]
	PE								
	PI								
	SMs								
GPLs	PC 34:1	Breast cancer (luminal, HER2+, and triple-negative)	Tissue	X*	–	X	–	–	[52]
Palmitoyl carnitines, stearoyl carnitine GPLs, SLs	PC16:0/16:0	Breast cancer (MDA-MB-231 model)	Tissue	X*	–	X	–	–	[60]
	PC16:0/18:1								
	PC18:1/18:1								
	PC18:0/18:1								
	PC16:0/22:1								
	SMd18:1/16:0								
GPLs	PI18:0/20:4	Breast cancer NS	Urine	–	X	X	–	–	[53]
GPLs	PS (18:1/18:1 18:2/18:0)	Breast cancer NS	Urine	X	–	X	–	–	[53]
GPLs	PI 18:0/18:1	Breast cancer NS	Tissue	X*	–	X	X	–	[54]
	PI 18:0/20:3								
GPLs	PCs	Breast cancer (mammary epithelial and breast cancer )	Cell lines	X	X	X	X	–	[58]
	PI 22:5/18:0								
	PI 18:0/18:1								
GPLs	PS 18:0/20:4	Breast cancer NS	Cell lines	X	–	–	X	X	[59]
	PI 18:0/20:4								
	PC 18:0/20:4								
GPLs	PIs	Breast cancer NS	Tissue	X	–	X	–	–	[115]
	PEs								
	PCs								
	LPCs								
GLs	TGs containing C18:1 fatty acyl chains	Breast cancer NS	Serum	–	X°	–	–	X	[61]
FAs	linoleic acid (C18:2)	Breast cancer NS	Serum	–	X°	–	–	X	[62]
GPLs	PS 18:0/18:1	Prostate cancer NS	Urine	X	–	X	–	–	[64]
	PS 16:0/22:6								
GPLs	PS 18:1/18:0	Prostate cancer NS	Urine	–	X	X	–	–	[64]
	PS 18:0/20:5								
GPLs	PI 18:0/18:1	Prostate cancer NS	Tissue	X*	–	X	–	–	[68]
	PI 18:0/20:3								
	PI 18:0/20:2								
GPLs	LPC 16:0/OH	Localized prostate cancer	Tissue	–	X*	X	–	–	[69]
	SM d18:1/16:0								
GPLs	LPC 16:0/OH	Localized prostate cancer	Tissue	–	X*	–	X	–	[69]
GPLs	PC 40:3	Newly diagnosed Prostate cancer	Serum	X§	–	X	–	–	[70]
	PC 42:4								
GPLs	PC 39:6	Prostate cancer NS	Serum	X§	–	X	–	–	[71]
FAs	FA 22:3								
GPLs	LPC 18:1	Colorectal cancer NS	Plasma	–	X	X	–	–	[72]
	LPC 18:2								
GPLs	PC/PE ratio	Colorectal cancer (pT 3 stage, various grades (G2, G3))	Cell lines	X	–	–	X	–	[73]
GPLs	PC 16:0/16:1	Colorectal cancer NS	Tissue	X	–	X	–	–	[74]
GPLs	PC 16:0/18:1	Colorectal cancer NS	Tissue	X*	–	X	–	–	[75]
	LPC 16:0								
	LPC 18:1								
GPLs	PE 38:6	Colorectal cancer liver metastasis	Tissue	X*	–	X	–	–	[77]
	PE 40:4								
FAs	n-3 PUFAs	Colorectal cancer NS	Red blood cell	–	X	X	–	–	[78]
FAs	n-6-PUFA/n-3-PUFA	Colorectal cancer NS	Red blood cell	X	–	X	–	–	[78]
GPLs	LPC	Ovarian cancer NS	Plasma	X	–	X	–	–	[79]
GPLs	PC	Ovarian cancer NS	Plasma	–	X	X	–	–	[79]
	TG								
GLs	TGs 50:2 50:1	Epithelial ovarian cancer	Cell lines	X	–	–	X	–	[81]
	52:2 54:4 54:3								
GPLs	PC 32:3	Ovarian cancer NS	Tissue	X	–	X	–	–	[80]
	PC 34:1								
	PC 36:2								
GPLs	LPA 16:0	Ovarian cancer NS	Plasma	X	–	X	–	–	[82]
	LPA 20:4								
GPLs	LPA	Ovarian cancer and other gynecological cancers	Serum/Plasma	X	–	X	–	–	[83,84,86]
GPLs	LPA 16:0	Ovarian cancer and other gynecological cancers	Plasma	X	–	X	–	–	[85]
	LPA 18:2								
	LPA18:1								
	LPA18:0								
	LPI 16:0								
	LPI 18:0								
	LPI 20:4								
GPLs	LPA	Benign and malignant ovarian cancer	Plasma	X	–	X	X	–	[87]
GPLs	LPA	Benign and malignant ovarian cancer	Plasma	X	–	X	–	–	[88]
GPLs	Plasmalogen phospatidylethanol, PC, plasmalogen PC, SM and LPC	Benign and malignant ovarian cancer		X	–	X	–	–	[89]
SLs	Ceramides species (C16:0 and C24:1)	Metastatic pancreatic cancer	Tissue/Plasma	X	–	X	X	–	[91]
SLs	C18:0	Metastatic pancreatic cancer	Tissue/Plasma	–	X	X	–	–	[91]
	C20:0								
	C22:0								
	C24:0								
	C24:1								
SLs	C16:0	Metastatic pancreatic cancer	Tissue/Plasma	X	–	X	X	–	[91]
	C20:0								
	C22:0								
	C24:0								
	C24:1								
GPLs	LPA	Pancreatic cancer PANC-1 cells	Cell lines	X	–	X	–	–	[94]
FAs	MUFA	Pancreatic cancer NS	Plasma	X	–	X	–	–	[95]
GPLs	PC16:0/18:1	Gastric cancer NS	Tissue	X	–	X	–	–	[97]
GPLs	LPC 16:0	Gastric cancer NS	Tissue	–	X	X	–	–	[97]
GPLs	PS 18:0/18:1	Bladder Cancer NS	Tissue	X	–	X	–	–	[100]
GPLs	PI 18:0/20:4	Bladder cancer	Tissue	X	–	X	–	–	[100]
GPLs	PS 18:0/18:1	Bladder cancer (Model of human invasive bladder cancer)	Tissue	X	–	X	–	–	[101]
GPLs	PG 18:1/18:1	Bladder cancer (Model of human invasive bladder cancer)	Tissue	X	–	X	–	–	[101]
GPLs	PI 16:0/18:1	Bladder cancer (Model of human invasive bladder cancer)	Tissue	X	–	X	–	–	[101]
GPLs	PI 18:0/18:1	Bladder cancer (Model of human invasive bladder cancer)	Tissue	X	–	X	–	–	[101]
–	PS 18:1/18:1	Bladder cancer (Model of human invasive bladder cancer)	Tissue	X	–	X	–	–	[101]
GPLs	Octanoylcarnitine LPC 16:1 Decanoylcarnitine	Esophageal cancer (ESCC)	Plasma	X	–	X	–	X	[105]
GPLs	PC 16:0/16:1	Esophageal cancer (OSCC)	Tissue	X	–	X	–	–	[107]
GPLs	PC 18:1/20:4	Esophageal cancer (OSCC)	Tissue	–	X	X	–	–	[107]
GPLs	PS	Esophageal cancer (ESCC)	Plasma	X	–	X	–	–	[106]
	PA								
	PC								
	PI								
	PE								
GLs SLs	PE (P-16:0e/0:0) ganglioside GM3 (d18:1/22:1) sphinganine C17 SMd18:0/16:1(9Z)	Kidney cancer NS	Serum	X	–	X	–	–	[112]
GPLs STLs Gls	PC	Kidney cancer NS	Tissue	X	–	X	–	–	[114]
	Plasmalogens								
	Cholesterol esters								
	TGs								
GPLs	PE	Kidney cancer NS	Tissue	–	X	X	–	–	[114]
FAs	Unsaturated FAs								
GPLs	PL	Kidney cancer NS	Tissue	X	–	X	–	–	[55]
	PE 36:1								
	PC 38:4								
	PC 36:2								
	PC 32:0								
GPLs	PE 34:2	Kidney cancer NS	Tissue	–	X	X	–	–	[55]
	PE 36:4								
	PE 38:4								
	PC 34:1								
	PC34:2								
	PC 36:4								
	PI36:4								
GPLs	PI18:0/20:4	Kidney cancer NS	Tissue	X	–	X	–	–	[116]
	PI22:4/18:0								
	PS18:0/18:1								
	PG18:1/18:1								
FAs	FA12:0	Kidney cancer (Human papillary renal carcinoma)	Tissue	–	X	X	–	–	[116]
GPLs	PC 16:0/18:1	Thyroid cancer (Thyroid papillary cancer)	Tissue	X	–	X	–	–	[118]
	PC 16:0/18:2								
SLs	SMd18:0/16:1	Thyroid cancer (Thyroid papillary cancer)	Tissue	X	–	X	–	–	[118]
GPLs	PC 34:1	Malignant and benign thyroid cancer	Tissue/serum	X	–	X	X	–	[119]
	PC 36:1								
	PC 32:0								
GPLs	PA 36:62	Malignant and benignant thyroid cancer	Tissue/serum	X	–	X	X	–	[119]
	PA 36:3								
	PA 38:4								
	PA 38:5								
	PA 40:5								
